# From generative-AI feedback to sport skill learning: the roles of perceived competence and autonomous motivation in university physical education

**DOI:** 10.3389/fpsyg.2026.1965551

**Published:** 2026-09-30

**Authors:** Jiehao Dong, Zhen Zhang

**Affiliations:** College of Physical Education, Anyang Normal University, Anyang, Henan, China

**Keywords:** autonomous motivation, generative artificial intelligence, perceived competence, sport skill learning, university physical education

## Abstract

**Introduction:**

Personalized feedback can support motor learning while also increasing reliance on external guidance. This study evaluated a structured personalized generative-artificial-intelligence (GenAI) feedback package in university physical education, focusing on immediate sport-skill acquisition, 14-day delayed retention, perceived competence, and autonomous motivation.

**Methods:**

In a two-arm randomized trial, 240 undergraduates completed an 8-week basketball unit and received either constrained written GenAI feedback generated from structured performance indicators or conventional oral instructor feedback matched for curriculum, practice dose, technical rubric, and feedback timing. Free-throw accuracy and blinded technique quality were assessed at baseline (T0), immediately after instruction (T1), and 14 days later without feedback (T2). Technique quality and shooting accuracy at T2 were co-primary endpoints with Holm control. Perceived competence and autonomous motivation were measured during the intervention, and mixed-model, binomial, robustness, and bootstrap indirect-effect analyses were conducted.

**Results:**

The GenAI-feedback package produced larger T0-T1 gains in shooting accuracy (differential gain = 6.7 percentage points, 95% CI [4.3, 9.1], d = 0.61) and technique quality (adjusted difference = 1.74 points, 95% CI [1.14, 2.34], d = 0.70). At T2, adjusted between-condition differences remained 6.1 percentage points for accuracy and 1.63 points for technique quality; both co-primary criteria were met after Holm correction. The GenAI condition also showed higher mid-intervention perceived competence and autonomous motivation. Serial indirect effects were statistically consistent with the proposed competence-to-autonomous-motivation sequence, but alternative parallel and reversed-order models were also compatible with the data.

**Discussion:**

The findings support the complete structured GenAI-feedback package relative to an active instructor-feedback package, not an isolated AI-specific effect. The 14-day result supports delayed retention over the tested interval rather than durable long-term learning, and the competence-motivation sequence should be interpreted as theory-consistent statistical evidence rather than a confirmed causal chain.

## 1 Introduction

A striking paradox in technology-supported physical education is that the same personalization that makes feedback useful can also make learners increasingly dependent on it. A correction tailored to a learner's current movement may clarify error, make progress visible, and focus the next attempt; yet if an external system continually diagnoses what is wrong and what to do next, improved performance during supported practice need not imply stronger independent regulation. This tension is especially relevant as generative artificial intelligence (GenAI) enters sport and physical-education settings. Studies increasingly connect AI-supported training and technology-enhanced instruction with technical and psychological outcomes, but technology use itself remains an insufficient explanation of motor learning (Zhang and Sun, 2026; Zhao et al., 2026).

The dependence problem is not new. Motor-learning research on augmented feedback has long examined guidance effects, feedback frequency, learner control, and the distinction between temporary performance facilitation and retention after support is removed (Salmoni et al., 1984; Wulf and Lewthwaite, 2016). The theoretical novelty of GenAI therefore cannot rest on rediscovering feedback dependence in a new technological setting. What GenAI adds is a different *feedback-construction capability*: from a common set of structured performance indicators, a generative system can synthesize several observations, prioritize one problem for a particular learner, explain why that problem matters, translate it into a concise corrective cue, and formulate a next-block target. Conventional augmented-feedback systems typically display scores, trajectories, videos, or predefined messages; GenAI can adapt the composition and explanation of the feedback episode while the underlying rubric and timing remain fixed. The relevant question is whether this adaptive synthesis improves learning when embedded within a bounded instructional architecture, not whether generative technology is intrinsically superior to conventional feedback.

University physical education provides a useful setting for that question because instructors often manage many learners with different starting levels while still needing to preserve opportunities for independent practice. Recent university PE research indicates that smartphone-based kinematic feedback and other structured digital supports can improve motor and motivational outcomes (Zhang, 2026; Müller and Wagner, 2026; Treschman et al., 2024). However, a personalized generative workflow also changes several learner-facing features at once: source, written vs. oral presentation, standardization, explanation depth, interface use, and reflection on the next action. The present experiment consequently treats the intervention as a *structured personalized GenAI-feedback package*. Any randomized difference is a package effect relative to an active instructor-feedback package; it cannot identify the isolated causal contribution of AI source, personalization, modality, novelty, expectancy, or the action-intention task.

The psychological account centers on perceived competence within self-determination theory (SDT). Perceived competence concerns the experience of effectiveness and growing mastery (Ryan and Deci, 2000). Corrective feedback can influence that experience because it changes how learners interpret success, failure, and the controllability of improvement. In sport and PE, competence-supportive feedback has been linked to motivational outcomes, and longitudinal evidence connects need-supportive instruction with perceived competence and intrinsic motivation (Kruse et al., 2024; Li and Fu, 2026). A structured feedback episode may therefore be useful when it turns a vague sense of “doing it wrong” into bounded information about what changed, what remains problematic, and what can be attempted next.

The focus on perceived competence is intentionally selective rather than exhaustive. The intervention also contained features that could plausibly support autonomy, including non-controlling wording, an actionable target, and a learner-written action intention. Autonomy-need satisfaction was not independently manipulated or included as a mediator in the present model. Instead, perceived competence was chosen as the focal proximal appraisal because technical corrective feedback directly communicates whether the learner can understand and improve task performance, whereas *autonomous motivation* captures the quality of regulation with which the learner subsequently engages in practice. Thus, the study does not claim that competence is the only SDT process involved; autonomy-supportive features remain part of the bundled intervention and a plausible unmeasured pathway.

A second distinction concerns acquisition vs. retention. Successful performance while assistance is available is not equivalent to motor learning. A no-feedback retention test asks whether a performance advantage remains after the external source of correction is withdrawn. The present 14-day test is therefore interpreted as *delayed retention over the tested interval*. It is informative about whether the observed advantage was restricted to assisted performance, but it is not evidence of durable long-term maintenance or transfer.

Accordingly, the study asks three questions: (RQ1) Does the structured personalized GenAI-feedback package produce greater immediate acquisition and 14-day delayed retention than conventional instructor feedback matched for curriculum, practice dose, rubric, and feedback timing? (RQ2) Is assignment to the GenAI-feedback package associated with higher perceived competence and autonomous motivation during learning? (RQ3) Are the data statistically consistent with the theory-proposed sequence from feedback condition to perceived competence, autonomous motivation, and subsequent sport-skill performance, while acknowledging that the two mediators were measured at the same Week-4 occasion? This framing treats the personalization paradox as the phenomenon, the competence–motivation sequence as one measured explanatory pathway, and bounded support as the design condition, without assuming that any residual condition effect reveals a separate causal route.

## 2 Theoretical background and hypothesis development

### 2.1 Generative-AI feedback beyond conventional augmented feedback

Feedback is a principal informational resource through which learners convert repeated movement attempts into more stable motor control. In conventional physical education, feedback may take the form of demonstration, verbal correction, knowledge of results, or knowledge of performance. Digital systems extend this repertoire by returning performance-relevant information rapidly; recent augmented-reality and video-feedback work, for example, links structured feedback with motor and motivational outcomes (He and Wei, 2025; Niznikowski et al., 2025; Abdelkafi et al., 2025). These findings establish that feedback design matters, but they also make clear that guidance effects and feedback dependence predate GenAI.

The theoretically distinctive affordance examined here is *adaptive synthesis under a fixed rubric*. The GenAI system did not acquire new sensory information that the instructor lacked, nor did it provide unrestricted conversation. It received a standardized set of performance indicators and converted them into a prioritized explanation, one corrective cue, and one next-block target. Relative to conventional augmented feedback, the novelty therefore lies in how multiple structured observations can be composed into an individualized explanatory message while the technical evidence base and feedback schedule are constrained. This capability may be useful in large classes, but it also increases the possibility that learners outsource interpretation to the system.

Motor-learning research distinguishes temporary performance changes under guidance from learning that remains detectable when guidance is removed (Salmoni et al., 1984). The immediate expectation is therefore that the structured personalized GenAI-feedback package can improve acquisition relative to the active instructor-feedback package, while delayed retention is required to determine whether the advantage extends beyond the supported instructional period.

H1. Students assigned to the structured personalized GenAI-feedback package will demonstrate greater improvement in immediate sport-skill performance than students receiving conventional instructor feedback.

### 2.2 Self-determination theory and the selective role of perceived competence

SDT provides the central psychological framework for examining why the feedback package may be associated with learning beyond the technical information itself. The theory distinguishes autonomy, competence, and relatedness as basic psychological needs, and recent AI-supported education research has used need-related processes to explain variation in learning motivation (Wang et al., 2026). The present model focuses on perceived competence because corrective feedback directly communicates whether performance discrepancies are understandable and improvable. A learner can miss the same number of shots as a peer yet interpret the experience differently depending on whether the cause of error is legible and the next correction appears attainable.

The intervention also incorporated non-controlling language, an observable next-step target, and a learner action intention. These features could plausibly contribute to autonomy-need satisfaction, expectancy, reflective engagement, or other processes. They were not independently randomized, and autonomy-need satisfaction was not measured. The model therefore does not claim that competence is the exclusive psychological mechanism. Instead, competence is treated as the focal feedback-proximal appraisal, while autonomous motivation represents the quality of subsequent engagement. This selective specification is intended to test a parsimonious SDT-consistent pathway rather than a complete model of all psychological consequences of the package.

A structured feedback episode may strengthen perceived competence by reducing ambiguity around failure: one priority error is identified, connected to the current movement pattern, and translated into a concrete next action. Recent PE research similarly suggests that support structures can shape motivation through internal resources such as self-efficacy and coping, while emerging GenAI studies indicate that motivational consequences depend on whether technology is experienced as supporting rather than controlling learner agency (Hao et al., 2025; Shao and Wang, 2026; Ahmed and Sultan, 2026). The relevant prediction is therefore about the structured feedback experience rather than AI source itself.

H2. Students assigned to the structured personalized GenAI-feedback package will report higher perceived competence during sport-skill learning than students receiving conventional instructor feedback.

### 2.3 Perceived competence and autonomous motivation

Within SDT, autonomous motivation refers to engagement that is experienced as self-endorsed because the activity is interesting, personally valued, or meaningfully connected to one's goals. This distinction is relevant to sport-skill learning because repeated practice requires learners to continue acting despite misses, fatigue, and temporary plateaus. Recent evidence suggests that teacher feedback may operate partly through expectancy and value processes even when direct performance effects vary (Chen et al., 2026).

Perceived competence can change the psychological meaning of repeated effort. When learners experience their actions as increasingly effective, an unsuccessful attempt may be interpreted less as evidence of inability and more as information about a controllable route toward mastery. In the present theory, competence is therefore the proposed experience through which technically relevant feedback acquires motivational meaning. Recent basketball and PE evidence illustrates how proximal psychological states can bridge instructional design and later performance (Ma et al., 2026).

Because perceived competence and autonomous motivation were both measured at Week 4, the proposed ordering cannot be established temporally within that occasion. The hypothesis therefore concerns a theory-consistent conditional association, not a claim that the measurement schedule proves competence causes later motivation. Alternative parallel and reversed-order specifications are used as sensitivity analyses to assess whether the observed data uniquely favor the proposed sequence.

H3. Higher perceived competence during sport-skill learning will be associated with higher autonomous motivation after baseline motivational differences and treatment condition are taken into account.

### 2.4 Autonomous motivation and subsequent sport-skill performance

Motor learning depends on repeated, effortful interaction with task constraints. Learners must attend to relevant movement information, tolerate unsuccessful attempts, apply corrective cues, and continue practice long enough for a more stable coordination pattern to emerge. Autonomous motivation is theoretically important because it may increase the likelihood that learners engage in these processes willingly rather than merely complete the minimum activity required by the class. Technology use in PE has been linked to fitness and learning outcomes partly through engagement, reinforcing the idea that motivational involvement can bridge instructional support and performance (Cui et al., 2024).

The present account does not treat motivation as a substitute for technical information. A motivated learner can still practice an ineffective movement pattern. Instead, autonomous motivation is expected to be associated with how corrective information is used across repeated attempts: carrying a correction forward, re-engaging after misses, and continuing deliberate practice when the external source of advice is unavailable.

H4. Higher autonomous motivation during the intervention will be associated with better subsequent sport-skill performance after baseline performance, perceived competence, treatment condition, and relevant participant characteristics are taken into account.

### 2.5 A theory-consistent statistical sequence from feedback to learning

The preceding arguments motivate the sequence *feedback condition*→*perceived competence*→*autonomous motivation*→*subsequent performance*. The randomized condition precedes both Week-4 mediators, and the Week-4 measures precede T1 and T2 motor outcomes. However, the two mediators were measured at the same occasion and were not randomized. The serial model therefore evaluates whether the observed covariance structure is statistically consistent with the proposed SDT sequence; it does not confirm a serial causal mechanism.

This distinction also changes how any remaining direct condition effect should be interpreted. A residual condition coefficient after including the mediators indicates only that the measured competence–motivation pathway does not account for the full randomized package effect. It cannot by itself identify a separate “informational route.” The remaining effect could reflect technical explanation, feedback modality, expectancy, novelty, reflective action planning, autonomy support, or other measured and unmeasured components of the bundled intervention. Parallel and reversed-order mediation models are therefore reported as alternative specifications rather than treated as tests of a uniquely established causal chain.

H5. Assignment to the structured personalized GenAI-feedback package will show an indirect association with post-intervention sport-skill performance that is statistically consistent with the theory-proposed sequence perceived competence → autonomous motivation.

### 2.6 Scaffolding rather than substitution as a design boundary

The proposed pathway is not expected to characterize every form of generated feedback. Advice that is technically invalid, overly broad, controlling, or excessively frequent may weaken the learner's role in error detection and correction. Diagnostic specificity, actionability, technically valid inputs, bounded timing, and opportunities to practice without receiving a new answer define the intended scaffold in the present intervention.

These features are design properties of the package, not independently manipulated moderators. The current two-arm experiment therefore cannot establish that specificity, actionability, or support fading are the active causal ingredients. Likewise, the GenAI condition differs from the conventional condition in feedback source, written vs. oral format, standardized structure, individualized explanation, interface use, and a learner action-intention step. The appropriate causal estimand is the effect of the complete structured GenAI-feedback package relative to the active instructor-feedback package. Factorial or dismantling designs are required to isolate any one component.

### 2.7 Immediate acquisition and 14-day delayed retention

Improvements observed while corrective information is available may reflect successful use of temporary guidance rather than stable learning. Recent work distinguishing acquisition, retention, and transfer reinforces the value of delayed no-feedback assessment when the objective is motor learning rather than temporary performance enhancement (Wang et al., 2025). This issue is particularly relevant for GenAI because highly accessible personalized feedback could increase reliance on external diagnosis.

The T2 test therefore functions as a delayed-retention assessment under support withdrawal. If the intervention advantage were restricted to assisted performance, substantial attenuation would be expected once technical feedback was removed. If a between-condition difference remains, the result indicates that a higher level of skill is still detectable after the 14-day interval. Such persistence is compatible with delayed retention but does not establish durable long-term maintenance, transfer, or psychological internalization.

H6. The between-condition advantage associated with the structured personalized GenAI-feedback package will remain evident on the 14-day no-feedback delayed-retention test; indirect effects through perceived competence and autonomous motivation are expected to remain directionally consistent with the post-intervention model.

## 3 Materials and methods

### 3.1 Research design and participants

The study was conducted as a two-arm, parallel-group randomized controlled trial embedded in an eight-week university physical-education unit. The two conditions were (a) a structured personalized GenAI-feedback package and (b) conventional instructor feedback. Both groups completed the same basketball curriculum, practice volume, testing schedule, technical rubric, and scheduled feedback frequency. The randomized contrast was interpreted at the package level: the GenAI condition combined generated written feedback, standardized structure, individualized explanation, interface use, and a brief learner action-intention step, whereas the active comparison used instructor-delivered oral feedback in the conventional course format. The experiment therefore estimates the effect of the complete GenAI-feedback package relative to the complete instructor-feedback package, not the independent causal effect of AI source or personalization alone.

Assessments were conducted at baseline (T0), during Week 4 for the psychological process measures, immediately after the intervention (T1), and 14 days after the final instructional session (T2). T2 was conducted without GenAI or instructor technical feedback and served as the delayed-retention assessment. The two T2 outcomes—blinded technique quality and free-throw accuracy—were treated as co-primary delayed-retention endpoints in the inferential framework used for this report. A positive primary result required both endpoints to favor the GenAI-feedback package after Holm correction across the two co-primary tests at a familywise two-sided α = 0.05. T1 technique quality and T1 accuracy were key secondary acquisition endpoints, and psychological outcomes and mediation analyses were explanatory outcomes.

An a priori recruitment target of 240 undergraduates (120 per condition) was set before recruitment. Under a conservative two-group approximation with a two-sided α = 0.05, 120 participants per condition provides approximately 87% power to detect a standardized difference of *d* = 0.40; even after 15% attrition, the corresponding power remains above 80% (Faul et al., 2009). Participants were recruited from required university PE courses. Eligibility criteria were age 18–24 years, enrollment in the selected basketball PE course, no current musculoskeletal injury limiting normal participation, and no formal competitive basketball training during the preceding 3 years. Students who were members of university or professional basketball teams were excluded to reduce ceiling effects.

The trial was implemented across four intact course sections taught by two basketball instructors. Individual randomization occurred within course section after T0 assessment using a computer-generated 1:1 sequence, with balancing by sex and baseline shooting performance. The allocation sequence was generated and retained by a researcher who did not deliver the intervention, and assignment was released only after baseline testing. Because participants necessarily knew whether feedback came from the GenAI interface or an instructor, participant blinding was not feasible. Outcome assessors responsible for video-based technique ratings were blinded to condition, time point, participant identity, and study hypotheses.

The trial was not prospectively registered, and no public protocol or externally timestamped statistical analysis plan was available. Accordingly, this revision does not use “pre-specified” or “preregistered” to characterize analyses. The endpoint hierarchy, covariate set, and sensitivity analyses are reported transparently as the analytic framework used for the present report and should not be interpreted as independently verifiable prospective registration.

### 3.2 Sport skill task and instructional setting

Basketball free-throw shooting was selected because it is common in university PE, can be standardized across repeated assessments, and permits simultaneous measurement of outcome success and movement quality. All assessments were conducted on regulation indoor courts using the same basket height, free-throw distance, and ball specification. Before each test, participants completed a standardized 10-min warm-up followed by five familiarization shots that were not scored. The formal test comprised 30 free throws arranged in three blocks of 10 with a 60-s seated interval between blocks. No technical feedback was provided during T0, T1, or T2.

Accuracy was recorded as the number of successful shots out of 30 and was also expressed as a percentage for descriptive reporting. Technique quality was derived from standardized frontal and sagittal video recordings and a five-domain scoring rubric covering lower-limb loading and extension, trunk and shoulder alignment, elbow/forearm alignment, release coordination, and follow-through consistency. Two cameras or smartphones mounted on fixed tripods reproduced the same position, frame rate, distance, and height at each assessment. Video files were anonymized and randomized before rating.

Two trained basketball instructors who did not deliver the intervention independently scored the assessment videos. A calibration session preceded formal scoring, and inter-rater reliability was evaluated with a two-way random-effects intraclass correlation coefficient. The mean of the two raters' total scores was used as the technique-quality outcome.

### 3.3 Technique-quality rubric development and scoring

Each of the five technique domains was scored from 1 to 5, yielding a total technique-quality range from 5 to 25. The anchors were designed to distinguish a substantial and inconsistent deviation (score 1), a partially correct but inconsistent pattern (score 3), and a technically aligned and consistently reproduced pattern (score 5), with scores 2 and 4 representing intermediate performance. Domain-specific anchors are summarized in Table 1.

**Table 1:** Technique-quality rubric and principal scoring anchors.

Domain	Score 1 anchor	Score 3 anchor	Score 5 anchor
Lower-limb loading and extension	Minimal or poorly timed knee/hip loading; force transfer visibly disrupted	Loading and extension present but timing or amplitude is inconsistent	Coordinated loading and extension with smooth upward force transfer
Trunk and shoulder alignment	Marked lateral/rotational deviation from target line	Generally aligned but recurrent small deviations across attempts	Stable trunk with shoulders consistently aligned to the target line
Elbow/forearm alignment	Elbow repeatedly displaced from the shooting line	Alignment intermittently correct but variable	Elbow and forearm consistently organized beneath/through the ball
Release coordination	Release poorly synchronized with extension; variable release point	Basic sequence present with intermittent timing inconsistency	Release consistently synchronized with whole-body extension at a stable point
Follow-through consistency	Follow-through absent, abbreviated, or highly variable	Appropriate follow-through appears on some but not all attempts	Full and repeatable wrist/finger finish maintained toward the target

Before the trial, six university basketball/PE specialists reviewed the rubric for relevance, clarity, and observability. Item-level content-validity indices ranged from 0.83 to 1.00 and the scale-level average content-validity index was 0.93. The wording and observable anchors were refined in a pilot scoring exercise with 24 students who were not included in the randomized sample. These procedures were used to support content validity before inter-rater reliability was evaluated in the trial videos.

### 3.4 Intervention structure and classroom implementation

Both conditions completed 16 instructional sessions over eight weeks, with two 60-min sessions per week. Each session included 10 min of warm-up, 10 min of standardized whole-class instruction, 30 min of structured shooting practice, and 10 min of consolidation. Core content progressed from stance and lower-limb force generation to upper-limb alignment, release coordination, follow-through, and integration under mild task variability. The same lesson objectives and practice counts were used in both conditions. Table 2 summarizes the matched instructional features and the components that differed between the two feedback packages.

**Table 2:** Matched instructional features and components that differed between feedback packages.

Feature	Personalized GenAI-feedback package	Conventional instructor-feedback package
Curriculum and practice dose	Identical 8-week curriculum; matched practice blocks and attempt targets	Identical 8-week curriculum; matched practice blocks and attempt targets
Feedback timing	Three scheduled 60–90-s episodes per session	Three scheduled 60–90-s episodes per session
Technical evidence base	Current block accuracy, five rubric indicators, and recent within-student trend	Current block accuracy and the same five rubric indicators
Source and modality	GenAI-generated individualized written message shown on a tablet	Instructor-delivered individualized oral correction
Structure	One strength, one priority correction, one explanation, one actionable cue, one next-block target	Brief instructor-selected priority correction and cue in conventional course format
Reflection/interface	One-sentence learner action intention entered after the message	No digital interface or required written action intention
Access outside window	No additional GenAI technical feedback	No additional individualized technical feedback beyond routine safety/clarification

During the 30-min structured practice period, students completed three focal practice blocks and received one feedback episode after each block. The feedback window was limited to approximately 60–90 s. Both conditions used the same five-domain rubric and the immediately preceding practice block as the technical evidence base. Safety instructions, demonstrations, and routine classroom management were identical across groups.

Both randomized conditions were represented within each course section. To reduce treatment contamination, participants practiced in designated lanes and used separate feedback stations during the scheduled feedback windows. GenAI tablets were accessible only to the assigned participant, and control participants received their technical feedback at an instructor station. Students were asked not to view or exchange condition-specific feedback until completion of T2. Independent fidelity observers sampled 20% of sessions and recorded scheduled-feedback delivery, unscheduled condition-specific coaching, and cross-condition feedback exposure. No cross-condition technical-feedback exchange was documented in the observed sessions. The classroom structure was handled from the study design rather than from a *post-hoc* decision based on the observed intraclass correlation. Because the trial contained only four intact course sections, course section was entered as a fixed design factor in the primary models rather than attempting to estimate a section-level random-effect variance from four units. The classes were taught by two basketball instructors; an instructor-level random effect was not estimated because two higher-level units do not provide a stable basis for variance-component estimation. Thus, the treatment-effect model did not add or remove classroom terms according to whether clustering happened to appear “non-negligible.”

To reduce instructor effects, the same instructional team used standardized lesson plans across sections and received protocol training on technical-feedback criteria, timing, and limits on unscheduled coaching. Session-level fidelity checklists recorded content coverage, approximate practice volume, completed feedback episodes, and protocol deviations.

### 3.5 Personalized generative-AI feedback condition

The GenAI condition used a constrained workflow rather than unrestricted conversation with a general-purpose chatbot. OpenAI GPT-5.6 Sol was used as the fixed generative model throughout the intervention and was accessed through the study-controlled interface. After each focal practice block, a structured performance record was created for the learner. The input contained the anonymized participant code, successful shots in the immediately preceding block, five rubric-based movement indicators, and a short within-student trend comparing the current and previous block. No names, health information, grades, or unrelated personal data were included. Model identity, input schema, system prompt, output format, and generation configuration were held constant across participants and sessions.

The five movement indicators were obtained in real time by basketball-trained research assistants from direct observation of the focal block. One assistant recorded the number of successful shots and assigned one 1–5 score for each technique domain; the interface calculated the within-student trend automatically and assembled the structured input. Assistants completed two protocol-training sessions and a calibration exercise before data collection. Human input was therefore required for observation and six structured entries per feedback episode, but not for composing the individualized narrative. Median manual entry time was 24 s (IQR 19–31 s) per episode. This distinction is important for scalability: the system automated synthesis and wording, not the underlying technical observation.

A fixed system prompt instructed the model to act as a formative basketball-skill feedback assistant. Output was constrained to approximately 70–100 words and had to contain five components in the same order: (a) one specific aspect performed well, (b) one highest-priority technical issue, (c) a brief explanation of why that issue affected the shot, (d) one actionable movement or external cue for the next block, and (e) one observable next-block target. The prompt prohibited more than one new correction at a time, medical or injury advice, comparisons with classmates, and controlling or derogatory language. When the structured evidence was insufficient, the system was instructed to state that no firm technical conclusion could be drawn rather than invent a diagnosis.

The interface displayed one generated response immediately after each scheduled block. Students read the message and recorded a one-sentence action intention describing what they would focus on in the next block. Students could not initiate free-form dialogue or request additional technical advice during practice. A teacher monitored safety and protocol adherence but did not routinely rewrite generated feedback. Unsafe, irrelevant, or technically inconsistent outputs were withheld and documented as protocol deviations. Model identity, system prompt, generation settings, structured inputs, and outputs were archived with the study materials.

### 3.6 Conventional instructor feedback condition

The control condition followed the same curriculum and received individualized instructor feedback at the same three scheduled points per session. The instructor observed the preceding practice block and used the same five-domain rubric available to the GenAI workflow. Feedback was delivered orally in the conventional course style and was limited to the same 60–90-s window. Instructors were asked to identify the highest-priority correction for the next block, provide a brief cue, and avoid extending the interaction into additional practice time.

The control condition was intentionally active rather than no-feedback. Instructor correction represented the realistic alternative to GenAI-supported feedback in university PE and reduced the possibility that a between-condition difference reflected feedback vs. no feedback. Routine encouragement, safety intervention, and clarification of task rules were permitted in both groups; condition-specific technical feedback was restricted to the scheduled episodes.

### 3.7 Measures

#### 3.7.1 Perceived competence

Perceived competence was assessed with the perceived-competence subscale of the Intrinsic Motivation Inventory (IMI), which has established use in sport and movement settings (McAuley et al., 1989). Items were adapted minimally so that the referent was the focal basketball-shooting task. Responses were recorded on a 7-point scale from 1 (*not at all true*) to 7 (*very true*), with negatively worded items reverse scored. The score was the mean of retained items, with higher values indicating a stronger experience of effectiveness and mastery. The measure was administered at T0, Week 4, and T1.

#### 3.7.2 Autonomous motivation

Autonomous motivation was measured using PE-specific behavioral-regulation items based on the Perceived Locus of Causality tradition (Goudas et al., 1994). Intrinsic regulation captured participation because the activity was enjoyable or interesting, whereas identified regulation captured participation because learning the skill was personally important or valued. Items were rated on a 7-point scale, and the primary autonomous-motivation index was the mean of intrinsic and identified regulation scores rather than a global score that mixes autonomous and controlled regulation (Li and Chang, 2026). The measure was administered at T0, Week 4, and T1.

The Week-4 competence and autonomous-motivation scores were used in the explanatory mediation analyses. Both followed repeated exposure to randomized condition and preceded T1/T2 motor outcomes, but they were measured at the same occasion. Their serial ordering was therefore theory-based rather than temporally demonstrated.

#### 3.7.3 Sport-skill acquisition and delayed retention

Sport-skill learning was represented by free-throw accuracy and blinded technique quality. T0 characterized baseline ability, T1 represented immediate acquisition, and T2 represented 14-day delayed retention under no-feedback conditions. The two T2 outcomes were the co-primary delayed-retention endpoints. Technique quality captures movement organization but depends on expert scoring; shooting accuracy is behaviorally direct but contains binomial sampling variation across 30 attempts. Their joint use reduces dependence on a single indicator.

#### 3.7.4 Manipulation checks and covariates

Perceived personalization and perceived actionability were assessed at Week 4 and T1 to verify that the two packages produced different learner-facing feedback experiences. They were not entered as additional mediators because neither was independently randomized. Observed differences therefore serve as manipulation checks rather than evidence that personalization or actionability alone caused the outcome effect.

The analysis covariates were age, sex, previous organized basketball experience, baseline weekly physical-activity frequency, baseline skill, and the corresponding baseline psychological score where relevant. Prior GenAI experience was recorded descriptively and examined in a sensitivity interaction because familiarity might affect novelty or credibility. Covariates were not expanded after inspection of treatment effects. Table 3 summarizes the assessment schedule and analytical role of the principal variables.

**Table 3:** Assessment schedule and analytical role of principal variables.

Measure	T0	Week 4	T1	T2/analytical role
Free-throw accuracy	Yes	–	Yes	Yes; co-primary delayed-retention endpoint; key secondary acquisition endpoint at T1
Blinded technique quality	Yes	–	Yes	Yes; co-primary delayed-retention endpoint; key secondary acquisition endpoint at T1
Perceived competence	Yes	Yes	Yes	–; focal feedback-proximal psychological variable
Autonomous motivation	Yes	Yes	Yes	–; quality-of-regulation psychological variable
Perceived personalization	–	Yes	Yes	–; manipulation check
Perceived actionability	–	Yes	Yes	–; manipulation check
Demographics/prior experience	Yes	–	–	–; descriptors and analysis covariates

### 3.8 Procedure

At T0, consenting participants completed demographic and prior-experience items, the psychological measures, and the standardized shooting test. Randomization occurred after baseline testing. During the first intervention session, both groups received the same explanation of course objectives, shooting rubric, feedback schedule, and assessment procedures. Participants in the GenAI condition additionally received a brief orientation to the feedback interface; the orientation did not provide extra basketball technique instruction.

Across Weeks 1–8, the two conditions followed the matched curriculum. The Week-4 psychological assessment was administered after a scheduled class but before the final practice block of that week so that it reflected accumulated intervention exposure without being collected during the final post-test. Session attendance, completed practice blocks, feedback exposure, and protocol deviations were recorded. Participants who missed a session remained in their assigned condition and could continue in the trial; no unscheduled feedback dose was added.

Within 3 days after the final instructional session, participants completed T1 under standardized no-feedback conditions. The post-test questionnaire followed the motor test. Fourteen days later, participants returned for T2. No experimental feedback was delivered between T1 and T2, and participants were asked not to use the study interface during that interval. The T2 protocol reproduced the T1 warm-up, familiarization, shot count, camera placement, and rest periods.

All digital records were stored under anonymized participant codes. The identity key was held separately from videos, questionnaires, and GenAI interaction logs. Only de-identified performance variables were provided to the generative system.

### 3.9 Statistical analysis

Analyses were organized into co-primary delayed-retention outcomes, key secondary acquisition outcomes, explanatory psychological/process analyses, and sensitivity analyses. Descriptive statistics, baseline balance, distributional characteristics, and missingness were examined first. All randomized participants remained assigned to their original group for the intention-to-treat (ITT) estimand; participants were not excluded from the analysis population because of non-adherence or missing follow-up data.

#### 3.9.1 Measurement model and longitudinal invariance

Internal consistency was evaluated with McDonald's ω and Cronbach's α. A two-factor confirmatory factor analysis (CFA) distinguished perceived competence from autonomous motivation. Because both constructs were measured at T0, Week 4, and T1, longitudinal measurement invariance was then evaluated using a correlated two-factor longitudinal CFA with the same item residuals allowed to correlate across occasions. Configural, metric, and scalar models were compared sequentially. Changes of |ΔCFI| ≤ 0.010 and ΔRMSEA ≤ 0.015 were treated as evidence that the additional equality constraints did not materially worsen fit. Composite-score comparisons and mediation analyses were interpreted only after at least scalar invariance was supported.

#### 3.9.2 Skill outcomes and multiplicity

For technique quality, linear mixed-effects models included fixed effects for condition, time, Condition × Time, and course section, with a participant-level random intercept. Planned contrasts estimated T0–T1 acquisition differences and adjusted between-condition contrasts at T2. Percentage-based linear mixed models were also used for free-throw accuracy because percentage-point differences are directly interpretable and the observed distributions were not concentrated near 0% or 100%. Because each accuracy score arose from 30 binary shots, a binomial generalized linear mixed model (GLMM) with logit link was added as a robustness analysis using successes and failures as the binomial numerator/denominator and the same condition, time, section, and participant structure. Baseline-adjusted ANCOVA models at T1 and T2 provided additional robustness checks.

#### 3.9.3 Model assumptions and diagnostics

The linear mixed-effects models assume approximately normal conditional residuals, approximately constant residual variance, and conditional independence after the participant-specific random intercept and fixed design terms are taken into account. These assumptions were evaluated with Q–Q plots of conditional residuals, residual-vs.-fitted plots, standardized residuals, and influence diagnostics. For the binomial GLMM, model convergence, boundary behavior, and overdispersion were examined. These diagnostic checks were used to assess model adequacy; they were not used to decide whether classroom structure should be represented, which followed the study design described above.

The familywise type-I error rate for the two co-primary T2 endpoints was controlled with Holm's procedure at two-sided α = 0.05. The primary trial conclusion was positive only if both co-primary endpoints favored the GenAI-feedback package after that adjustment. The two T1 acquisition endpoints formed a key secondary family and were also evaluated with Holm correction. Psychological secondary-outcome families were controlled using the false-discovery rate. Explanatory mediation estimates were reported with bootstrap confidence intervals and were not part of the co-primary success criterion.

#### 3.9.4 Psychological associations and alternative mediation specifications

Condition differences in perceived competence and autonomous motivation were estimated after adjustment for their baseline values and analysis covariates. Subsequent-outcome models estimated the associations of Week-4 psychological variables with T1/T2 skill after adjustment for baseline skill, condition, and relevant baseline psychological values.

The primary explanatory serial model specified condition → Week-4 perceived competence → Week-4 autonomous motivation → skill outcome, with baseline competence, baseline motivation, and baseline skill in the relevant equations. Indirect effects used 5,000 non-parametric bootstrap resamples. Randomization supports causal inference for assignment to feedback package, but the mediator–mediator and mediator–outcome paths remain conditional associations. Because the mediators were concurrent at Week 4, two alternative specifications were examined: (a) parallel mediation with competence and autonomous motivation entered simultaneously and allowed to covary, and (b) reversed serial ordering with autonomous motivation preceding perceived competence. The purpose of these alternatives was to determine whether the proposed sequence was uniquely supported by the observed data, not to select a causal order from same-occasion measures.

#### 3.9.5 Missing data and ITT handling

Primary mixed models used all available repeated observations under maximum-likelihood estimation and retained participants according to randomized allocation. Missingness was summarized by condition and assessment occasion, including intermittent and monotone patterns. Multiple imputation by chained equations was used as an ITT sensitivity analysis with 50 imputed datasets. The imputation model included condition, course section, time, baseline and observed follow-up skill outcomes, perceived competence, autonomous motivation, age, sex, previous basketball experience, physical-activity frequency, attendance, feedback exposure, and prior GenAI use. Predictive mean matching was used for bounded continuous/scale variables and appropriate logistic models for binary variables; imputed shot counts were constrained to the observed 0–30 range. Estimates were pooled using Rubin's rules. The imputation analysis assumes data were missing at random conditional on variables included in the imputation model. Complete-case analysis was reported separately so that conclusions could be compared under a more restrictive missing-data strategy.

Additional robustness analyses examined protocol-compliant participants, attendance adjustment, feedback-exposure adjustment, and alternative course-section specification. Prior basketball experience and prior GenAI experience were examined separately as exploratory effect-modification checks rather than as determinants of the primary model structure. All tests were two-sided. Numerical estimates, standard errors, confidence intervals, Wald statistics, and *p*-values for the principal robustness specifications are reported in Supplementary Table 4 rather than summarized only as “materially similar.”

### 3.10 Ethical considerations

The study was reviewed and approved by the Ethics Committee of Anyang Normal University (approval number: ANU-2601128). All procedures involving human participants were conducted in accordance with the Declaration of Helsinki and applicable institutional requirements. Participation was voluntary and independent of course grading. All participants provided written informed consent before data collection and were informed of their right to withdraw without academic penalty. Video recordings, questionnaire responses, and GenAI interaction records were stored under coded identifiers, and only the minimum de-identified performance information required for personalized feedback was transmitted to the generative system.

## 4 Results

### 4.1 Participant flow, missingness, baseline comparability, and implementation fidelity

A total of 247 students were screened. Seven did not enter randomization because they either did not meet eligibility criteria (*n* = 4) or declined research participation (*n* = 3). The remaining 240 students completed T0 and were randomized equally to personalized GenAI feedback (*n* = 120) or conventional instructor feedback (*n* = 120). T1 motor-performance data were available for 234 participants (117 per condition), and 229 participants completed T2 (115 GenAI, 114 control). Across the two post-baseline motor assessments, 227 participants had complete T1 and T2 data; two missed T1 but returned for T2, seven completed T1 but missed T2, and four missed both post-baseline motor assessments. Thus, missingness was sparse and was not concentrated in one randomized condition. All 240 participants remained in the ITT analysis population and were analyzed according to original assignment. Occasion-specific questionnaire availability and the full missingness pattern are reported in Supplementary Table 1.

Participant flow is summarized in Figure 1.

**Figure 1:**
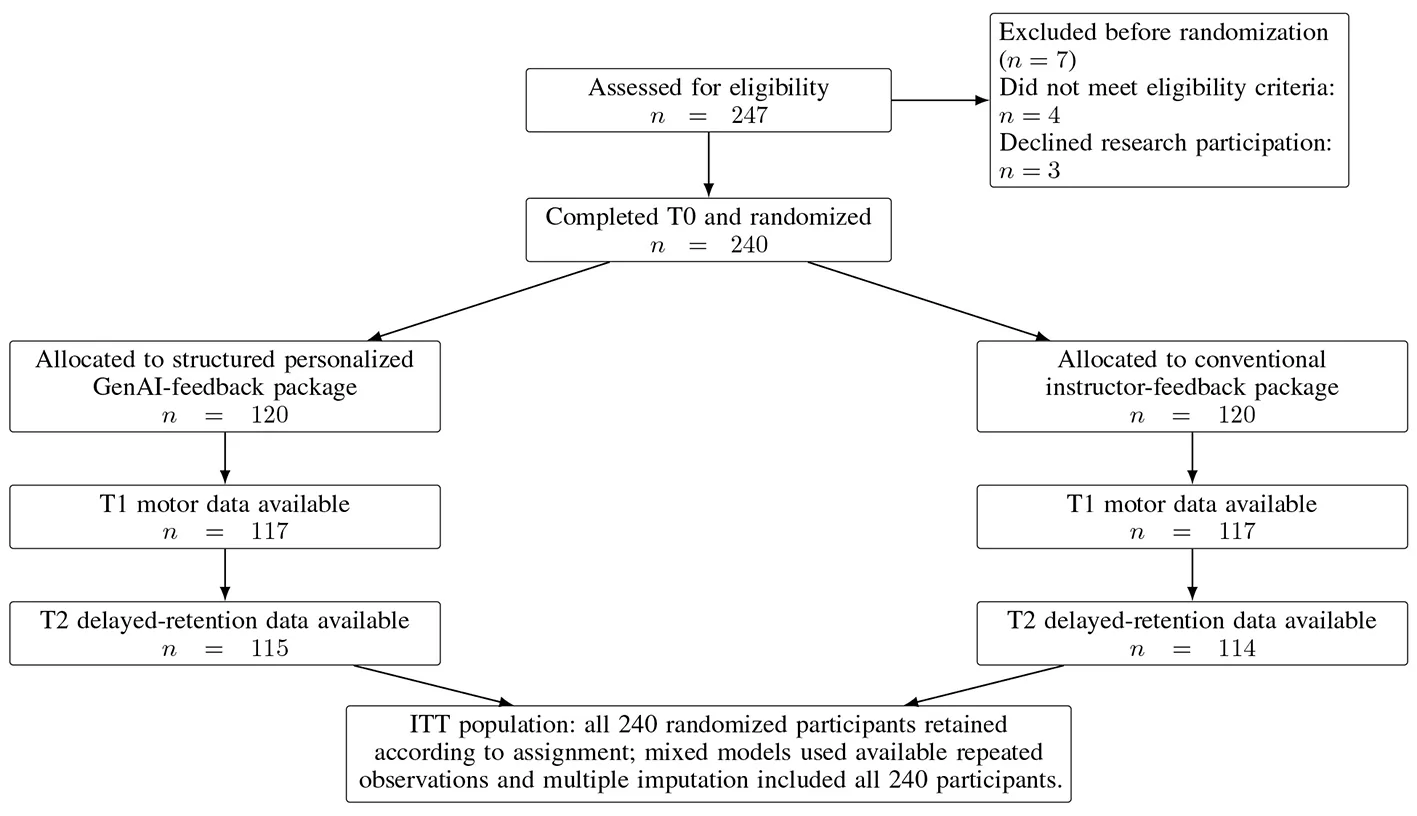
Participant flow through the randomized trial. All randomized participants remained in the intention-to-treat population and were analyzed according to allocation. Mixed-effects models retained available repeated observations; the multiple-imputation sensitivity analysis included all 240 randomized participants.

The randomized groups were closely comparable at baseline (Table 4). Absolute standardized mean differences were below 0.12 for the analysis variables. The two blinded technique raters demonstrated high agreement, ICC(2,2) = 0.91, 95% CI [0.88, 0.93].

**Table 4:** Baseline characteristics of the randomized sample.

Characteristic	GenAI feedback (*n* = 120)	Instructor feedback (*n* = 120)	Standardized difference
Age, years, *M* (*SD*)	20.11 (1.10)	20.08 (1.06)	0.03
Women, *n* (%)	64 (53.3)	62 (51.7)	0.03
Previous organized basketball experience, *n* (%)	26 (21.7)	25 (20.8)	0.02
Weekly physical-activity frequency, *M* (*SD*)	3.11 (1.21)	3.19 (1.18)	–0.07
Prior GenAI use, *n* (%)	101 (84.2)	99 (82.5)	0.05
Free-throw accuracy, %, *M* (*SD*)	43.2 (11.4)	43.6 (11.2)	–0.04
Technique-quality score, *M* (*SD*)	14.10 (2.62)	14.18 (2.58)	–0.03
Perceived competence, *M* (*SD*)	4.18 (0.78)	4.16 (0.81)	0.03
Autonomous motivation, *M* (*SD*)	4.62 (0.72)	4.58 (0.75)	0.05

Participants attended a mean of 15.1 of 16 sessions in the GenAI condition and 15.0 in the instructor condition. Mean completed focal feedback episodes were 45.2 and 44.9, respectively, of a maximum of 48. Independent fidelity observations indicated protocol-consistent delivery in 95.8% of observed GenAI episodes and 95.1% of observed instructor episodes. Three GenAI outputs were withheld because they did not satisfy the technical-consistency rule; these events represented less than 0.1% of generated feedback episodes. No cross-condition technical-feedback exchange was documented during the independently observed sessions.

### 4.2 Measurement properties, longitudinal invariance, and manipulation checks

The psychological measures showed satisfactory internal consistency across occasions. McDonald's ω ranged from 0.86 to 0.91 for perceived competence and from 0.85 to 0.90 for autonomous motivation; corresponding Cronbach's α values ranged from 0.84 to 0.90. A two-factor CFA distinguishing perceived competence from autonomous motivation fit the pooled questionnaire data well, χ^2^(34) = 54.7, *p* = 0.014, CFI = 0.982, TLI = 0.976, RMSEA = 0.050, 90% CI [0.025, 0.073], and SRMR = 0.035. The latent-factor correlation was 0.58.

Longitudinal CFA then tested whether the constructs operated comparably across T0, Week 4, and T1. The configural model showed good fit, and equality constraints on factor loadings produced negligible change in fit. Constraining item intercepts likewise resulted in ΔCFI and ΔRMSEA well within the stated criteria. Scalar invariance was therefore supported (Table 5), providing a basis for comparing composite scores and using the repeated constructs in the process analyses.

**Table 5:** Longitudinal measurement invariance for perceived competence and autonomous motivation across T0, Week 4, and T1.

Model	χ^2^	*df*	CFI	TLI	RMSEA	SRMR	ΔCFI	ΔRMSEA
Configural	421.8	306	0.976	0.969	0.041	0.044	–	–
Metric	443.6	326	0.975	0.970	0.040	0.047	–0.001	–0.001
Scalar	470.9	346	0.973	0.969	0.041	0.049	–0.002	0.001

The manipulation checks confirmed that the randomized packages produced different learner-facing feedback experiences without changing scheduled feedback frequency. At Week 4, perceived personalization was higher in the GenAI condition (*M* = 5.61, *SD* = 0.73) than in the instructor condition (*M* = 5.02, *SD* = 0.78), adjusted difference = 0.58, 95% CI [0.39, 0.77], *p* < 0.001, *d* = 0.78. Perceived actionability was also higher (*M* = 5.52, *SD* = 0.68 vs. *M* = 5.17, *SD* = 0.72), adjusted difference = 0.34, 95% CI [0.16, 0.52], *p* < 0.001, *d* = 0.50. The same directional pattern was observed at T1. Because neither feature was separately randomized, these checks verify treatment separation but do not identify a unique causal ingredient.

### 4.3 Immediate sport-skill acquisition

Observed outcome trajectories are shown in Table 6. Both conditions improved from baseline, with larger gains in the structured GenAI-feedback package. For free-throw accuracy, the percentage-based mixed model showed a Condition × Time interaction, *F*(2, 451.6) = 14.82, *p* < 0.001. From T0 to T1, accuracy increased by 18.6 percentage points in the GenAI condition and 11.6 points in the instructor condition. The model-estimated differential gain was 6.7 percentage points, 95% CI [4.3, 9.1], *p* < 0.001, corresponding to *d* = 0.61. Holm correction across the two T1 acquisition endpoints did not change this conclusion.

**Table 6:** Observed means for sport-skill and psychological outcomes across assessment occasions.

Outcome	Condition	T0 baseline	Week 4	T1 post-test	T2 delayed retention
Free-throw accuracy (%)	GenAI	43.2 (11.4)	–	61.8 (10.7)	58.9 (11.1)
	Instructor	43.6 (11.2)	–	55.2 (11.0)	52.7 (11.3)
Technique quality	GenAI	14.10 (2.62)	–	19.34 (2.44)	18.81 (2.51)
	Instructor	14.18 (2.58)	–	17.52 (2.53)	17.02 (2.59)
Perceived competence	GenAI	4.18 (0.78)	5.26 (0.73)	5.48 (0.70)	–
	Instructor	4.16 (0.81)	4.72 (0.80)	4.91 (0.82)	–
Autonomous motivation	GenAI	4.62 (0.72)	5.31 (0.69)	5.49 (0.68)	–
	Instructor	4.58 (0.75)	4.86 (0.77)	5.00 (0.76)	–

Values are unadjusted *M* (*SD*). Psychological measures were scored on 1–7 scales.

The binomial GLMM using successful vs. unsuccessful shots out of 30 produced the same substantive result. At T1, the adjusted odds of a successful shot were higher in the GenAI-feedback condition, OR = 1.40, 95% CI [1.18, 1.67], *p* < 0.001; the corresponding model-implied probability contrast was 6.5 percentage points. Thus, the result was not dependent on treating accuracy as a continuous percentage.

Technique quality showed the same directional pattern, Condition × Time *F*(2, 449.9) = 20.36, *p* < 0.001. Scores increased from 14.10 to 19.34 in the GenAI condition and from 14.18 to 17.52 in the instructor condition. The baseline-adjusted T1 difference was 1.74 points, 95% CI [1.14, 2.34], *p* < 0.001, *d* = 0.70. Together, the two key secondary acquisition endpoints supported H1 for the package-level comparison.

Figure 2 shows substantial individual overlap and heterogeneity despite a shift toward larger gains in the GenAI-feedback group on both objective outcomes. The joint-gain panel indicates that gains in accuracy generally co-occurred with gains in technique rather than reflecting a trade-off. The visible T2 separation is treated descriptively here; formal delayed-retention inference is reported below.

**Figure 2:**
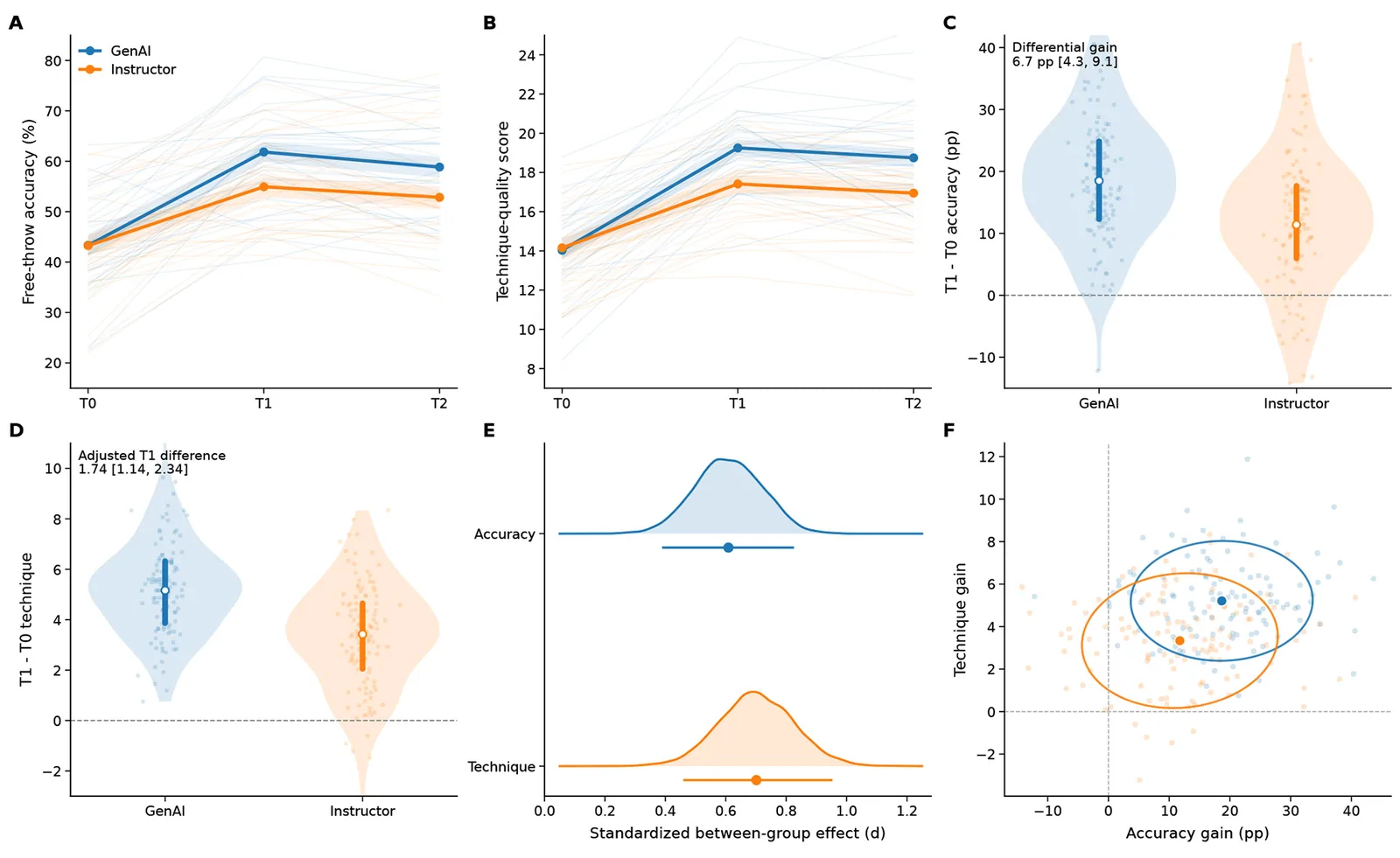
Sport-skill acquisition and performance gains under the structured GenAI-feedback package. **(A)** Free-throw accuracy across T0, T1, and T2. **(B)** Blinded technique-quality trajectories. **(C)** Distribution of individual T0–T1 accuracy gains. **(D)** Distribution of technique gains with the baseline-adjusted T1 contrast. **(E)** Standardized between-group effects. **(F)** Joint distribution of accuracy and technique gains. The figure describes trajectory and heterogeneity; inferential conclusions are based on the models reported in the text.

### 4.4 Perceived competence and autonomous motivation

Assignment to the structured GenAI-feedback package was associated with higher perceived competence during the intervention. After adjustment for T0 competence and analysis covariates, the Week-4 difference was *B* = 0.52, *SE* = 0.10, 95% CI [0.33, 0.71], *p* < 0.001, standardized β = 0.31. The difference remained evident at T1, adjusted *B* = 0.54, 95% CI [0.35, 0.73], *p* < 0.001. These findings support H2 as a randomized package-level effect on the measured competence outcome.

Week-4 perceived competence was positively associated with Week-4 autonomous motivation after adjustment for baseline motivation, baseline competence, condition, and analysis covariates, *B* = 0.44, *SE* = 0.06, 95% CI [0.32, 0.56], *p* < 0.001, standardized β = 0.46. Condition also retained an independent association with Week-4 autonomous motivation, *B* = 0.22, 95% CI [0.07, 0.37], *p* = 0.004. These coefficients support H3 as an association but cannot establish within-Week-4 temporal precedence.

Autonomous motivation was positively associated with subsequent objective outcomes. Controlling for baseline skill, baseline autonomous motivation, perceived competence, condition, and analysis covariates, a one-point increase in Week-4 autonomous motivation was associated with a 0.72-point higher T1 technique score, 95% CI [0.35, 1.09], *p* < 0.001, standardized β = 0.21, and 2.45 percentage points higher T1 shooting accuracy, 95% CI [0.85, 4.05], *p* = 0.003, standardized β = 0.18. These results are consistent with H4, but the mediator–outcome paths remain observational conditional associations. Figure 3 summarizes the psychological response profiles and the corresponding conditional associations with subsequent skill performance.

**Figure 3:**
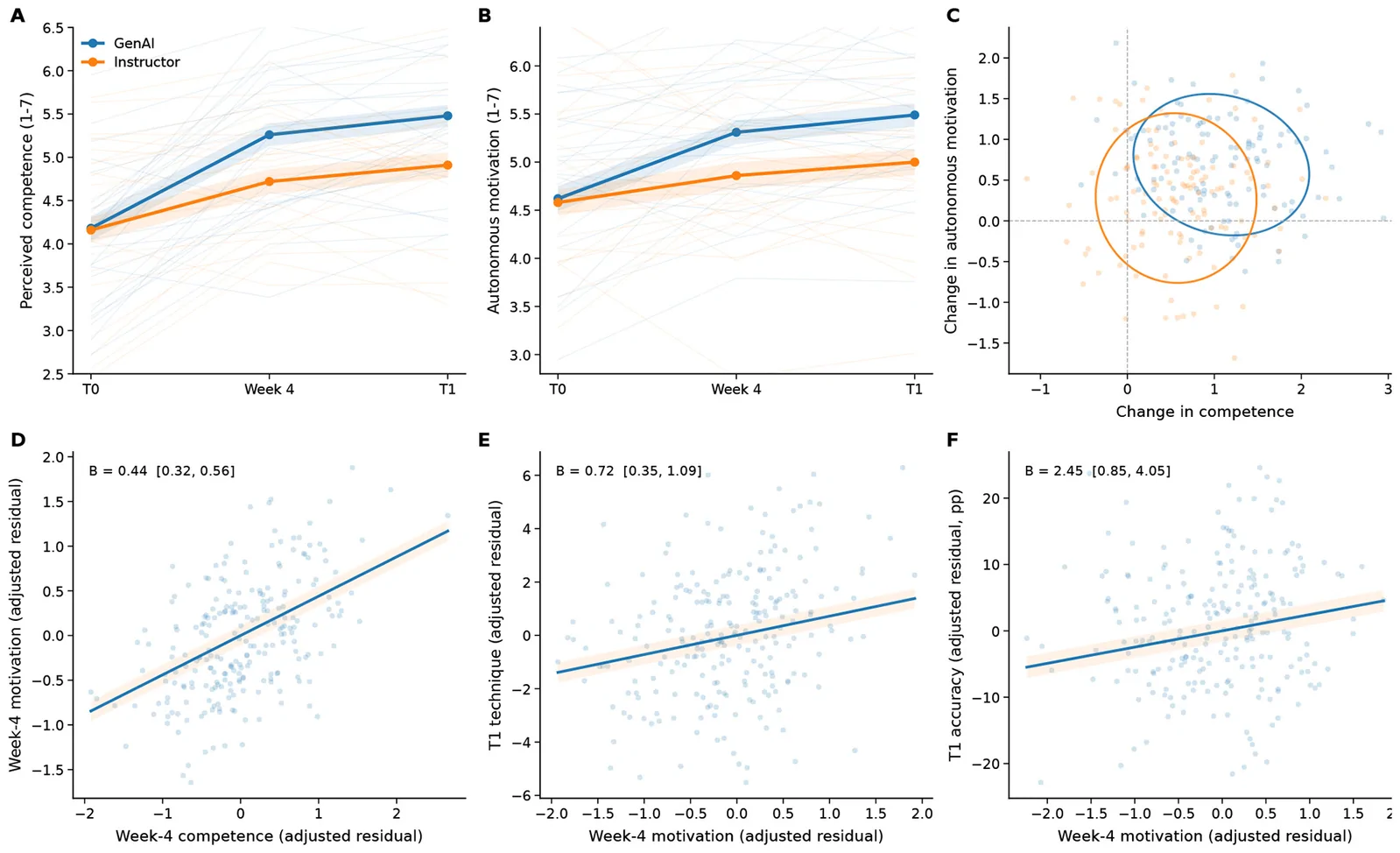
Psychological response profiles and conditional associations with subsequent skill performance. **(A)** Perceived competence from T0 to Week 4 and T1. **(B)** Autonomous motivation across the same occasions. **(C)** Joint T0–Week-4 changes. **(D)** Covariate-adjusted association between Week-4 competence and Week-4 autonomous motivation. **(E)** Association between Week-4 autonomous motivation and T1 technique quality. **(F)** Association with T1 free-throw accuracy. Panels D–F summarize conditional associations and are not interpreted as randomized mediator effects.

### 4.5 Theory-consistent indirect effects and alternative mediation specifications

The theory-proposed serial model was first estimated for T1 technique quality. The condition-to-competence path was positive (*a*_1_ = 0.52, *p* < 0.001), Week-4 competence was associated with Week-4 autonomous motivation (*d*_21_ = 0.44, *p* < 0.001), and autonomous motivation was associated with T1 technique quality (*b*_2_ = 0.72, *p* < 0.001). The serial indirect effect was *B* = 0.17, 95% bootstrap CI [0.07, 0.29], standardized indirect effect = 0.031. For T1 shooting accuracy, the corresponding serial indirect effect was 0.56 percentage points, 95% bootstrap CI [0.21, 1.03], standardized indirect effect = 0.024. These estimates are statistically consistent with H5, but they do not establish that competence temporally caused autonomous motivation because both mediators were measured at Week 4.

Alternative models clarified this limitation. In a parallel-mediation specification, both competence and autonomous motivation showed positive specific indirect associations with T1 outcomes when entered simultaneously. In the reversed serial model, the indirect sequence condition → autonomous motivation → perceived competence → outcome was also positive and its bootstrap interval excluded zero, although the point estimate was smaller than for the theory-proposed sequence. The data therefore did not uniquely identify mediator ordering. This pattern supports treating the competence-to-motivation sequence as theory-consistent rather than empirically confirmed. Numerical estimates are provided in Supplementary Table 3.

The direct condition effect remained detectable after the measured mediators were included. This residual does not demonstrate a separate informational pathway; it indicates that the measured competence–motivation variables account for only part of the package effect and leaves other bundled or unmeasured processes unresolved.

Table 7 summarizes the principal path estimates and indirect effects for the theory-proposed sequence. Figure 4 shows that the proposed serial indirect estimates were positive across both skill outcomes and both post-baseline occasions, but modest relative to the total condition effect. The serial estimate represented 9.8% of the total T1 condition effect for technique quality and 8.4% for accuracy. The appropriate inference is therefore partial statistical explanation by the measured competence–motivation sequence, not evidence of two independently demonstrated causal routes.

**Table 7:** Principal estimates for the theory-proposed indirect sequence.

Model path or effect	*B*	*SE*	95% CI	*p*
GenAI condition → Week-4 perceived competence	0.52	0.10	[0.33, 0.71]	<0.001
Week-4 perceived competence → Week-4 autonomous motivation	0.44	0.06	[0.32, 0.56]	<0.001
Week-4 autonomous motivation → T1 technique quality	0.72	0.19	[0.35, 1.09]	<0.001
Week-4 autonomous motivation → T1 shooting accuracy	2.45	0.81	[0.85, 4.05]	0.003
Serial indirect effect on T1 technique quality	0.17	–	[0.07, 0.29]	–
Serial indirect effect on T1 shooting accuracy	0.56	–	[0.21, 1.03]	–
Serial indirect effect on T2 technique quality	0.15	–	[0.05, 0.27]	–
Serial indirect effect on T2 shooting accuracy	0.49	–	[0.16, 0.94]	–

Indirect-effect confidence intervals are based on 5,000 bootstrap resamples.

**Figure 4:**
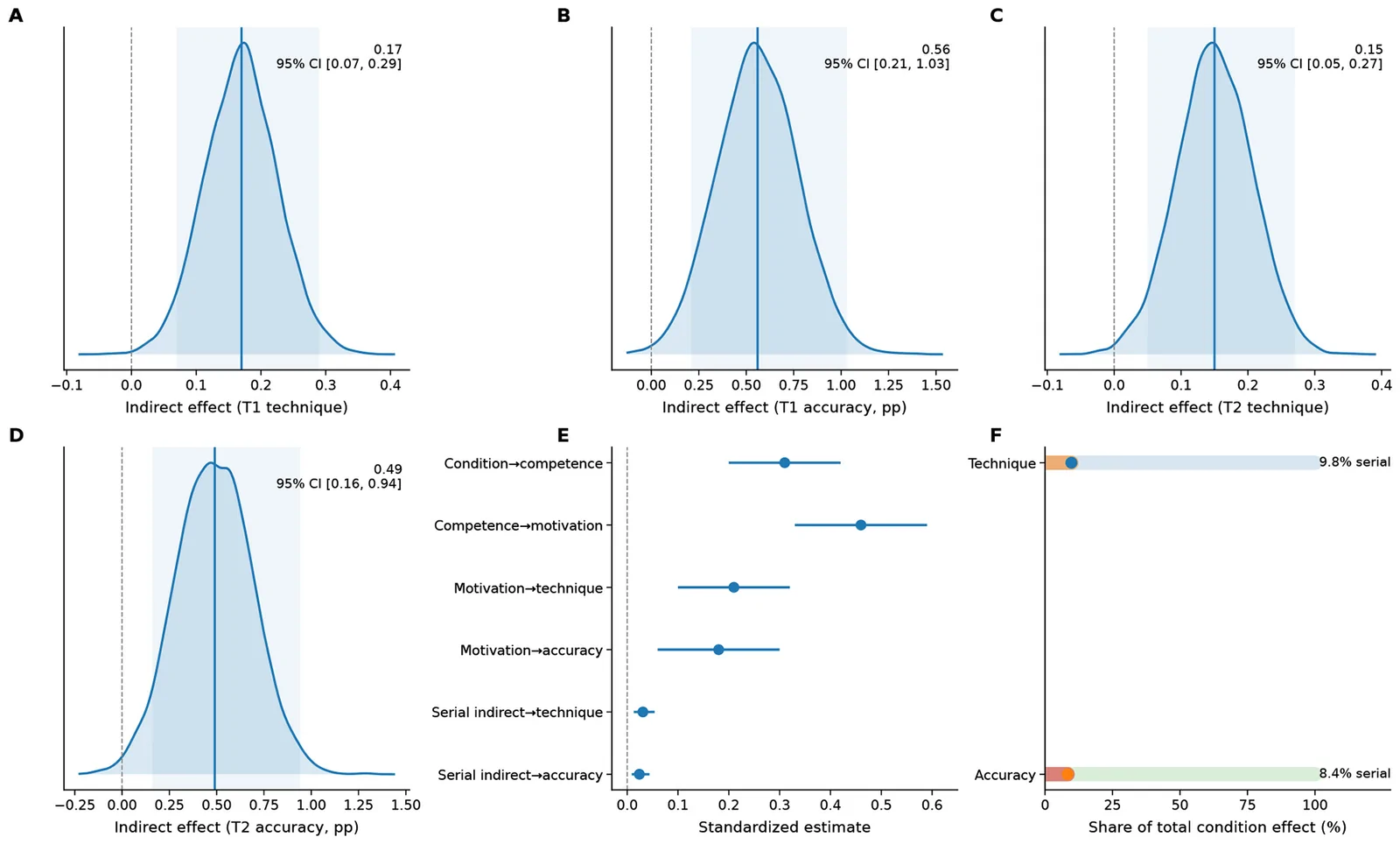
Indirect-effect profile across immediate acquisition and 14-day delayed retention. **(A–D)** Bootstrap distributions of the theory-proposed serial indirect effect for T1 technique quality, T1 accuracy, T2 technique quality, and T2 accuracy. **(E)** Standardized estimates for principal component paths and T1 indirect effects. **(F)** Proportion of the total T1 condition effect represented by the measured serial indirect sequence. The figure summarizes a theory-consistent statistical process pattern; it does not establish temporal ordering between the two Week-4 mediators or identify the meaning of the residual direct effect.

### 4.6 Co-primary 14-day delayed-retention outcomes

At T2, both groups showed modest declines from T1, but the GenAI-feedback group remained at a higher performance level. For shooting accuracy, the mean T1–T2 decline was 2.9 percentage points in the GenAI condition and 2.5 points in the instructor condition; the difference in decline was not statistically detectable, *B* = −0.41, 95% CI [-2.35, 1.53], *p* = 0.68. The baseline-adjusted T2 between-condition difference was 6.1 percentage points, 95% CI [3.7, 8.5], *p* < 0.001, *d* = 0.55. The binomial GLMM confirmed the same conclusion, OR = 1.36, 95% CI [1.14, 1.63], *p* = 0.001, with a model-implied probability contrast of approximately 6.0 percentage points.

Technique quality declined by 0.53 points from T1 to T2 in the GenAI condition and 0.50 points in the instructor condition; the difference in decline was not statistically detectable, *p* = 0.84. The adjusted T2 condition effect was 1.63 points, 95% CI [1.01, 2.25], *p* < 0.001, *d* = 0.64.

After Holm correction across the two co-primary T2 endpoints, both remained statistically significant in the direction favoring the structured GenAI-feedback package; the primary success criterion was therefore met. The retention result should be interpreted as a higher skill level remaining detectable 14 days after feedback withdrawal, not as evidence of slower forgetting, durable long-term learning, or transfer to a different task.

The theory-proposed serial indirect effect was directionally consistent at T2: *B* = 0.15, 95% bootstrap CI [0.05, 0.27] for technique quality and 0.49 percentage points, 95% CI [0.16, 0.94] for accuracy. These supportive process estimates remain subject to the same concurrent-mediator limitation as the T1 models.

### 4.7 Sensitivity analyses and hypothesis summary

The conclusions were stable across the principal robustness specifications. Baseline-adjusted ANCOVA, multiple imputation, complete-case analysis, protocol-compliant analysis, attendance adjustment, exposure adjustment, and alternative course-section adjustment all yielded package-effect estimates close to the primary mixed-model estimates. Diagnostic inspection of the principal linear mixed models did not indicate a material departure from conditional residual normality or homoscedasticity, and no single participant exerted disproportionate influence on the estimated package effects. The binomial GLMM converged without boundary warnings or material overdispersion and further showed that the accuracy conclusion did not depend on analyzing 30-shot success as a continuous percentage. Exploratory effect-modification checks for prior organized basketball experience and prior GenAI use did not show reliable Condition × Experience interactions for the principal T1/T2 outcomes (all interaction *p*>0.10); these checks are not interpreted as evidence of equivalence across experience strata.

Figure 5 summarizes the robustness diagnostics for the principal sport-skill outcomes. The robustness figure is therefore supplemented by direct numerical reporting rather than relying on visual overlap or phrases such as “materially similar.” Supplementary Table 4 reports the analysis population, model specification, point estimate, standard error, 95% confidence interval, Wald statistic, and *p*-value for each principal T1 and T2 robustness analysis. Table 8 summarizes the results for H1-H6 and their interpretive status.

**Figure 5:**
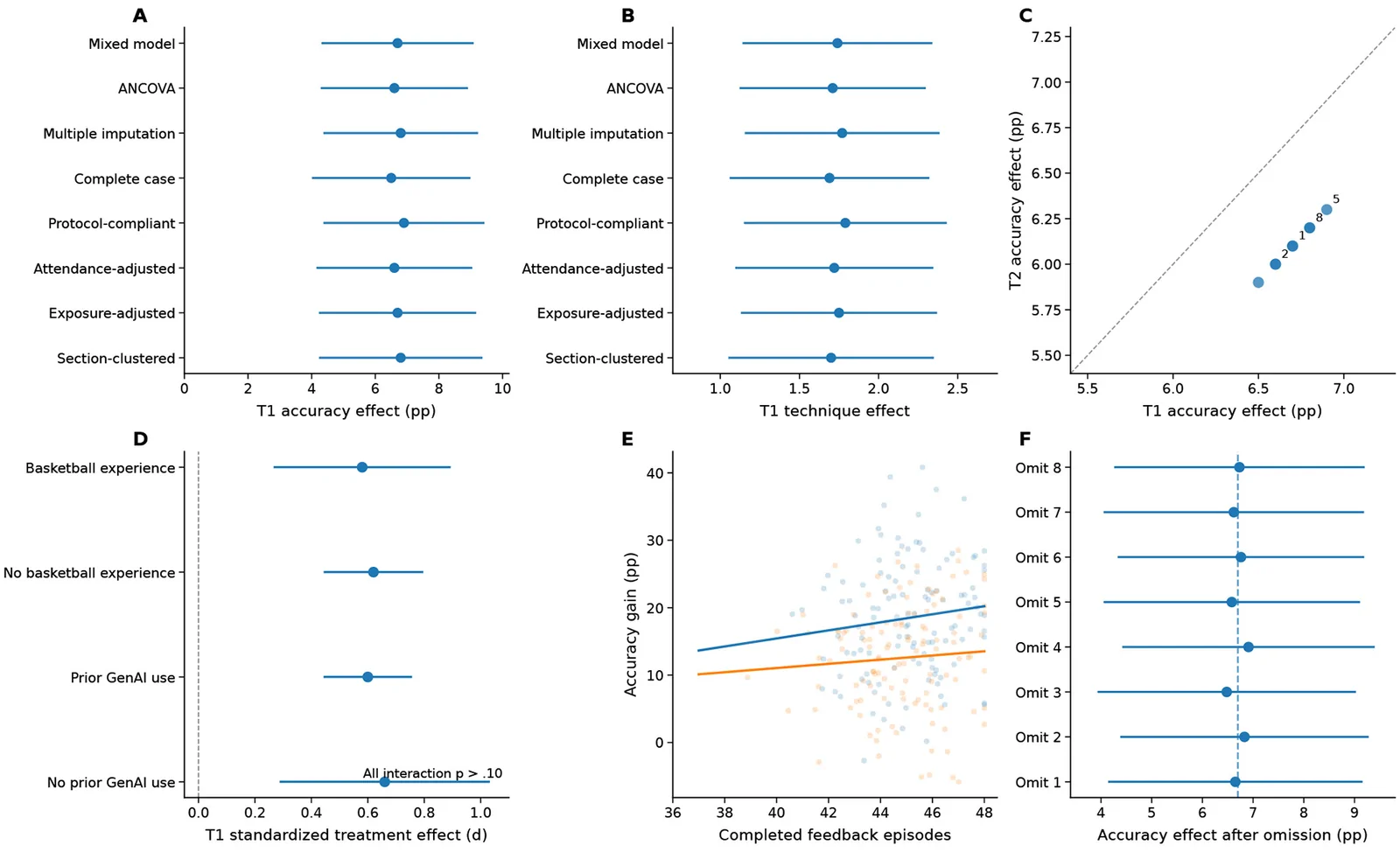
Robustness diagnostics for estimated package effects on sport-skill outcomes. **(A)** T1 free-throw accuracy across eight analytic specifications. **(B)** T1 technique-quality effects. **(C)** Paired T1/T2 accuracy estimates for representative specifications. **(D)** Standardized T1 effects by prior basketball and prior GenAI experience; interaction tests were non-significant. **(E)** Descriptive association between completed feedback episodes and accuracy gain. **(F)** Leave-one-specification-out diagnostic for T1 accuracy. Numerical estimates for the principal sensitivity specifications are given in Supplementary Table 4.

**Table 8:** Summary of hypothesis tests.

Hypothesis	Primary result	Conclusion
H1	Larger T0–T1 gains in shooting accuracy and blinded technique quality for the structured GenAI-feedback package.	Supported
H2	Higher perceived competence at Week 4 and T1 in the GenAI condition.	Supported
H3	Week-4 perceived competence positively associated with concurrent autonomous motivation after adjustment.	Supported as association
H4	Autonomous motivation positively associated with subsequent objective skill outcomes.	Supported as association
H5	Theory-proposed serial indirect effects excluded zero, but alternative models showed that mediator order was not uniquely identified.	Consistent, not causal proof
H6	Both co-primary T2 outcomes favored the GenAI-feedback package after Holm control.	Supported for 14-day retention

## 5 Discussion

### 5.1 Principal findings

The study examined a structured personalized GenAI-feedback package within an eight-week university basketball unit. Three findings organize the interpretation. First, assignment to the GenAI-feedback package was associated with larger immediate gains in free-throw accuracy and blinded technique quality than an active instructor-feedback package matched on curriculum, practice dose, rubric, and feedback timing. Second, the GenAI condition showed higher perceived competence and autonomous motivation during the intervention, and the observed process data were statistically consistent with the theory-proposed competence-to-motivation sequence. Third, both co-primary performance outcomes remained higher 14 days after feedback withdrawal. The binomial analysis of successful shots and the numerical sensitivity analyses reproduced the main performance conclusions.

The randomized comparison must nevertheless remain package-level. The GenAI condition differed in source, written modality, standardized structure, individualized explanation, interface use, and an action-intention task. Expectancy, novelty, modality, reflective engagement, autonomy support, and other bundled features were not independently manipulated. The findings therefore do not identify an AI-specific causal effect or the independent effect of personalization. They estimate what happened when this complete feedback architecture was used instead of the conventional instructor-feedback architecture under otherwise matched instructional conditions.

### 5.2 What GenAI adds beyond the established guidance problem

The present contribution does not depend on claiming that feedback dependence is new. Guidance effects, feedback frequency, and retention after augmented feedback are established motor-learning concerns. The GenAI-specific question is narrower: can a generative system dynamically *compose* structured evidence into one learner-specific explanation and next-step cue while remaining bounded by a common rubric and schedule? In the present workflow, the model did not create new performance evidence; it transformed human-entered indicators into an individualized message. The theoretical advance is therefore a test of adaptive explanatory synthesis within an established guidance-vs.-learning problem, not a transfer of an old dependency effect to a fashionable technology.

This distinction also explains why the package effect is pedagogically meaningful even though it is not AI-specific. Conventional instructor feedback and the GenAI package represent two realistic feedback architectures. The trial shows that the latter architecture produced a different learning pattern under the tested conditions; dismantling studies are still needed to determine whether the difference arose from generative synthesis, written modality, standardization, reflection, expectancy, or interactions among these components.

### 5.3 Perceived competence, autonomous motivation, and what the mediation can support

Perceived competence was higher in the GenAI-feedback condition during the intervention. One plausible interpretation is that the package reduced ambiguity around error by connecting current performance to one prioritized correction and an attainable next action. That interpretation is consistent with SDT and with the manipulation checks showing greater perceived personalization and actionability. It remains a plausible explanation rather than an independently manipulated causal ingredient because diagnostic specificity, actionability, and expectancy were not factorially separated.

The selective focus on perceived competence also requires restraint. The package used non-controlling wording, next-step goals, and a learner-written action intention, all of which could plausibly influence autonomy-need satisfaction or reflective engagement. Autonomy-need satisfaction was not measured. Consequently, the present data do not show that competence is the only or necessarily dominant SDT process. Perceived competence is better understood as the focal measured appraisal that was theoretically proximal to corrective feedback, whereas autonomous motivation represented the quality of engagement with subsequent practice.

The mediation results are consistent with this theoretical sequence but do not confirm a serial causal mechanism. Perceived competence and autonomous motivation were measured together at Week 4, so temporal precedence between them was not established. The reversed serial model also yielded a positive indirect estimate, and the parallel model showed positive specific indirect associations. These alternative specifications make the inferential boundary explicit: the observed data support a competence–motivation process configuration, but they do not uniquely identify the direction of influence between the two concurrent mediators.

The remaining direct condition effect should be interpreted equally cautiously. It demonstrates that the measured mediators do not statistically account for the full randomized package effect. It does not demonstrate a separate informational route. The unexplained portion could reflect technical explanation, modality, expectancy, novelty, action planning, autonomy-related processes, or other components that were not independently measured or manipulated.

### 5.4 Immediate acquisition and 14-day delayed retention

The delayed-retention findings are important because motor-learning research has long cautioned that augmented feedback can improve assisted performance without producing an advantage after support is removed (Akizuki et al., 2025). Here, both groups declined modestly from T1 to T2, and the rate of decline did not differ detectably. The key observation is therefore not slower forgetting in the GenAI group; it is that the group reached a higher level at T1 and that a higher level remained detectable 14 days later without experimental technical feedback.

This result is compatible with delayed retention over the tested interval and argues against an effect restricted entirely to feedback-assisted performance. It should not be described as strong evidence of durable long-term learning. A 14-day interval cannot establish maintenance over months, transfer to altered task constraints, or independent skill regulation across contexts. Those stronger claims require longer follow-up and transfer tests.

### 5.5 Theoretical implications

The first theoretical contribution is a more precise placement of GenAI within motor-learning theory. GenAI does not introduce the guidance problem; it changes the capacity to construct individualized feedback from structured evidence. The study therefore connects generative personalization to an established acquisition–retention framework while specifying the novel affordance as adaptive synthesis rather than simple feedback availability.

Second, the study identifies one measured psychological pathway that can be discussed without treating it as a confirmed mechanism. Assignment to the feedback package preceded changes in perceived competence, and competence and autonomous motivation were jointly related to later objective performance. The indirect effects indicate that this measured competence–motivation configuration accounts for part of the package effect. The alternative mediation results, however, prevent the data from establishing a unique serial order. The theoretical value lies in narrowing a plausible SDT process to be tested more causally in future work, not in declaring the process proven.

Third, the study treats support withdrawal as part of the theoretical test. A generative feedback architecture is educationally more compelling when performance remains detectable after the system is removed than when gains occur only while the system supplies answers. The present 14-day finding satisfies that limited criterion, while leaving longer-term maintenance, transfer, and optimal fading schedules open for direct study.

### 5.6 Implications for university physical education and sport training

The intervention is most plausibly useful in instructional settings with four characteristics: repeated low-risk motor practice, a stable technical rubric, frequent need for short individualized explanations, and sufficient digital infrastructure to deliver bounded feedback without interrupting practice. Large university PE classes fit this profile because instructors can define the technical standard while a system helps translate structured observations into concise individualized messages.

The workflow does not eliminate human judgment. In the present implementation, a trained human still observed the focal block, recorded shot success, and entered five rubric scores before the model generated the narrative. Instructors remained responsible for defining valid criteria, monitoring safety, interpreting unusual cases, and withholding technically inconsistent output. The generative system therefore automated synthesis and wording, not movement diagnosis itself. Claims that the approach reduces instructor workload would be premature without direct time-and-motion measurement. A more defensible implication is that it may *reallocate* work from repeatedly composing similar explanations toward validating performance indicators and supervising exceptions.

This division of labor also identifies a practical scalability boundary. If obtaining reliable movement indicators requires substantial expert observation, the bottleneck shifts upstream rather than disappearing. Future systems may reduce that cost through validated computer-vision measures or simpler learner-facing input, but those solutions introduce new validity and safety questions. Implementation studies should therefore report not only learning outcomes but also observation time, data-entry burden, device requirements, exception rates, and teacher oversight.

Finally, more feedback should not automatically be treated as better feedback. Bounded timing and no-feedback practice intervals preserve opportunities for learners to act on prior information without receiving a new diagnosis after every attempt. Future trials should manipulate fading schedules and learner control directly rather than inferring that the scaffold-vs.-substitution boundary was causally established by the current package.

### 5.7 Limitations and prioritized future directions

The limitations are best understood as three design boundaries rather than a list of unrelated flaws. *First, causal identification is limited to the randomized package contrast*. Source, modality, standardization, individualized explanation, interface use, action intention, expectancy, novelty, and possible autonomy support were bundled. The psychological mediators were measured rather than manipulated, and perceived competence and autonomous motivation were concurrent at Week 4. Thus, the trial supports a causal conclusion about assignment to the complete package, while the proposed psychological sequence and specific feedback ingredients remain explanatory hypotheses. The next priority is a dismantling or factorial design combined with temporally separated mediator measurement, including autonomy-need satisfaction and, where feasible, experimental manipulation of competence-relevant information.

*Second, implementation and scalability remain bounded by human input and the constrained system architecture*. Human observers supplied the performance indicators used for generation, and the present study did not directly quantify instructor workload saved or added. The results therefore should not be generalized to unrestricted consumer-chatbot use or interpreted as evidence that GenAI can substitute for professional observation. Future implementation studies should compare teacher time, input burden, technical error rates, automated vs. human indicator extraction, and the cost of safety oversight.

*Third, generalizability and temporal scope are limited*. The study examined one university PE context, one standardized basketball skill, and a 14-day delayed-retention interval. The design is well-suited to repeated standardized trials and blinded technique scoring, but it does not establish transfer to open skills, team tactics, high-risk environments, or longer-term maintenance. Priority replications should therefore use different motor tasks, longer follow-up intervals, and transfer tests that change distance, task constraints, or performance context.

These boundaries do not negate the randomized comparison; they define what the present design can and cannot establish. The next research step is not simply to make the GenAI system more complex, but to isolate which components matter, determine whether the psychological sequence holds when temporally separated, and test whether similar benefits persist when human input and support are progressively reduced.

## 6 Conclusion

This randomized trial compared a structured personalized GenAI-feedback package with conventional instructor feedback during an eight-week university basketball unit. The package produced larger immediate gains in free-throw accuracy and blinded technique quality, and both co-primary performance outcomes remained higher at a 14-day no-feedback delayed-retention assessment after multiplicity control. A binomial model of successes out of 30 shots and multiple sensitivity specifications supported the same performance conclusion.

Perceived competence and autonomous motivation were also higher in the GenAI condition, and the observed indirect effects were statistically consistent with the proposed competence-to-autonomous-motivation sequence. Because both mediators were measured at Week 4 and alternative models were also compatible with the data, the sequence should be interpreted as a theory-consistent statistical process rather than a confirmed serial causal mechanism. Likewise, the residual direct condition effect cannot identify a separate informational route.

The appropriate contribution is therefore bounded. The study provides evidence for this complete feedback architecture relative to an active instructor-feedback package, not for an isolated AI-specific effect. The 14-day result supports delayed retention over the tested interval, not durable long-term learning. Future work should dismantle the bundled intervention, measure autonomy-related and competence-related processes at separated time points, quantify human workload, and test longer-term retention and transfer.

## Data Availability

The raw data supporting the conclusions of this article will be made available by the authors, without undue reservation.

## References

[B1] Abdelkafi H. Ghorbel A. Yaakoubi M. Kammoun M. M. Alsaeed M. I. Gharbi A. . (2025). The effect of video observation on the frequency, type, and accuracy of peer feedback in a long jump learning context. Front. Psychol. 16:1737033. doi: 10.3389/fpsyg.2025.1737033 41536537 PMC12796952

[B2] Ahmed A. Sultan A. (2026). Examining the impact of generative ai on student motivation and engagement: the mediating role of autonomy-support and autonomous motivation in education. Front. Psychol. 17:1852265. doi: 10.3389/fpsyg.2026.1852265 42453433 PMC13364909

[B3] Akizuki K. Takeuchi K. Yabuki J. Yamaguchi K. Yamamoto R. Kaneno T. (2025). Effects of self-control of feedback timing on motor learning. Front. Psychol. 16:1638827. doi: 10.3389/fpsyg.2025.1638827 41050791 PMC12491054

[B4] Chen J. Haw J. Yan Z. King R. B. (2026). The role of teacher feedback on students' motivation: a situated expectancy-value perspective. Front. Psychol. 17:1835077. doi: 10.3389/fpsyg.2026.1835077 42376155 PMC13312164

[B5] Cui Z. Song Y. Du X. (2024). Multilevel modeling of technology use, student engagement, and fitness outcomes in physical education classes. Front. Psychol. 15:1458899. doi: 10.3389/fpsyg.2024.1458899 39512567 PMC11540672

[B6] Faul F. Erdfelder E. Buchner A. Lang A.-G. (2009). Statistical power analyses using g*power 3.1: tests for correlation and regression analyses. Behav. Res. Methods 41, 1149–1160. doi: 10.3758/BRM.41.4.1149 19897823

[B7] Goudas M. Biddle S. J. H. Fox K. R. (1994). Perceived locus of causality, goal orientations, and perceived competence in school physical education classes. Br. J. Educ. Psychol. 64, 453–463. doi: 10.1111/j.2044-8279.1994.tb01116.x 7811633

[B8] Hao H. Zhu Q. Feng C. (2025). The impact of multiple supports on university students' physical education learning motivation: a dual analysis based on sem and fsqca. Front. Psychol. 16:1446317. doi: 10.3389/fpsyg.2025.1446317 40104420 PMC11913872

[B9] He X. Wei L. (2025). Real-time feedback enhances motor learning and motivation in youth team sports through augmented reality tools. Front. Psychol. 16:1661936. doi: 10.3389/fpsyg.2025.1661936 41426421 PMC12714985

[B10] Kruse F. Bühel S. Brühwiler C. (2024). Longitudinal effects of basic psychological need support on the development of intrinsic motivation and perceived competence in physical education: a multilevel study. Front. Psychol. 15:1393966. doi: 10.3389/fpsyg.2024.1393966 39035084 PMC11259123

[B11] Li K.-H. Chang C.-K. (2026). Agentic ai as a dual-role lecturer and teaching assistant: effects on learner autonomy, self-regulated learning, and intrinsic motivation in university-level efl education. Front. Psychol. 17:1847607. doi: 10.3389/fpsyg.2026.1847607 42427376 PMC13346227

[B12] Li Y. Fu N. (2026). Motivational and demotivational teaching styles in physical education: the role of basic psychological needs. Front. Psychol. 17:1839188. doi: 10.3389/fpsyg.2026.1839188 42344969 PMC13287065

[B13] Ma C. Liu L. Zhao Y. Gu Y. Li Z. (2026). Psychological readiness mediates performance transfer in constraints-led, game-based basketball training: evidence from a quasi-experimental study. Front. Psychol. 17:1735248. doi: 10.3389/fpsyg.2026.1735248 42369287 PMC13293939

[B14] McAuley E. Duncan T. Tammen V. V. (1989). Psychometric properties of the intrinsic motivation inventory in a competitive sport setting: a confirmatory factor analysis. Res. Quart. Exerc. Sport 60, 48–58. doi: 10.1080/02701367.1989.10607413 2489825

[B15] Müller J. Wagner I. (2026). Formative assessment of motor learning through digital tools in physical education: a systematic literature review. Eur. Phys. Educ. Rev. 32, 383–403. doi: 10.1177/1356336X251357207

[B16] Niznikowski T. Arnista P. Sadowski J. Mastalerz A. Romero-Ramos O. Fernandez-Rodriguez E. . (2025). Enhancing the learning of sports skills through verbal feedback. Front. Sports Active Liv. 7:1519365. doi: 10.3389/fspor.2025.1519365 40264934 PMC12011859

[B17] Ryan R. M. Deci E. L. (2000). Self-determination theory and the facilitation of intrinsic motivation, social development, and well-being. Am. Psychol. 55, 68–78. doi: 10.1037/0003-066X.55.1.68 11392867

[B18] Salmoni A. W. Schmidt R. A. Walter C. B. (1984). Knowledge of results and motor learning: a review and critical reappraisal. *Psychol*. etin 95, 355–386. doi: 10.1037/0033-2909.95.3.355 6399752

[B19] Shao H. Wang R. (2026). Generative ai an academic equalizer? The differential impact of ai-assisted learning on self-efficacy and intrinsic motivation among university students. Front. Psychol. 17:1779741. doi: 10.3389/fpsyg.2026.1779741 41982358 PMC13071819

[B20] Treschman P. Stylianou M. Brooks C. (2024). A scoping review of feedback in physical education: conceptualisations and the role of teachers and students. Eur. Phys. Educ. Rev. 30, 545–562. doi: 10.1177/1356336X241230829

[B21] Wang B. Tao T. Yuan Y. Guo W. (2025). Self-controlled feedback and behavioral outcomes in motor skill learning: a meta-analysis. Behav. Sci. 15:1291. doi: 10.3390/bs15091291 41009321 PMC12467369

[B22] Wang Y. Yang K. Han D. (2026). The influence of ai literacy on pre-service teachers' learning motivation: the mediating role of basic psychological needs. Front. Psychol. 17:1831770. doi: 10.3389/fpsyg.2026.1831770 42183496 PMC13194449

[B23] Wulf G. Lewthwaite R. (2016). Optimizing performance through intrinsic motivation and attention for learning: the optimal theory of motor learning. Psychon. Bull. Rev. 23, 1382–1414. doi: 10.3758/s13423-015-0999-9 26833314

[B24] Zhang D. (2026). Enhancing motor skill acquisition and autonomous motivation in university physical education: a randomized controlled trial of a steam-integrated approach. Front. Psychol. 17:1754141. doi: 10.3389/fpsyg.2026.1754141 42145549 PMC13176267

[B25] Zhang H. Sun J. (2026). The associations of ai assisted training on sport performance among student athletes based on a dual path chain mediation model with the moderating role of psychological adaptability. Front. Psychol. 16:1695704. doi: 10.3389/fpsyg.2025.1695704 41694761 PMC12894264

[B26] Zhao Y. Wang Y. Liu L. Ma C. Li Z. (2026). Ai-assisted and big-unit teaching enhance speed-skating performance through psychological mechanisms in adolescents: evidence from a three-arm intervention study. Front. Psychol. 17:1750654. doi: 10.3389/fpsyg.2026.1750654 41704415 PMC12907373

