# Supplementary material for: From generative-AI feedback to sport skill learning: the roles of perceived competence and autonomous motivation in university physical education
Source: Front Psychol. 2026 Sep 30;17:1965551. doi: 10.3389/fpsyg.2026.1965551 (PMC13661541; doi:10.3389/fpsyg.2026.1965551)
Supplement: Supplementary file 1 [file Data_Sheet_1.pdf]

Supplementary Material

SUPPLEMENTARY MATERIAL

Table S1. Supplementary Table S1. Missing-data pattern by randomized condition.

Data availability pattern	GenAI ($n = 120$)	Instructor ($n = 120$)	Total
Complete T1 and T2 motor outcomes	114	113	227
T1 missing, T2 observed	1	1	2
T1 observed, T2 missing	3	4	7
Both T1 and T2 motor outcomes missing	2	2	4
Week-4 psychological questionnaire available	118	118	236
T1 psychological questionnaire available	116	116	232

Note: All 240 randomized participants remained in the ITT population. Mixed models used available repeated observations.

Table S2. Supplementary Table S2. Binomial GLMM robustness analysis for free-throw accuracy.

Contrast	Odds ratio	95% CI	Model-implied probability difference	p
GenAI vs. instructor at T1, baseline-adjusted	1.40	[1.18, 1.67]	+6.5 percentage points	$< .001$
GenAI vs. instructor at T2, baseline-adjusted	1.36	[1.14, 1.63]	+6.0 percentage points	.001

Note: Binomial response = successful shots out of 30; logit link; participant random intercept; condition, time, Condition $\times$ Time, and course section included.

Table S3. Supplementary Table S3. Alternative mediation specifications for the two T1 outcomes.

Model specification	T1 technique quality, indirect effect [95% bootstrap CI]	T1 shooting accuracy, indirect effect [95% bootstrap CI]
Theory-proposed serial: condition $\rightarrow$ competence $\rightarrow$ autonomous motivation $\rightarrow$ outcome	0.17 [0.07, 0.29]	0.56 [0.21, 1.03]
Reversed serial: condition $\rightarrow$ autonomous motivation $\rightarrow$ competence $\rightarrow$ outcome	0.09 [0.01, 0.18]	0.28 [0.04, 0.62]
Parallel: specific indirect via competence	0.22 [0.09, 0.38]	0.58 [0.17, 1.13]
Parallel: specific indirect via autonomous motivation	0.16 [0.05, 0.30]	0.46 [0.13, 0.90]
Parallel: total indirect association	0.38 [0.20, 0.60]	1.04 [0.42, 1.78]

Note: Alternative models are sensitivity analyses for model dependence; concurrent Week-4 measurement prevents causal ordering of the mediators.

Table S4. Supplementary Table S4. Numerical robustness analyses for principal sport-skill effects.

Panel A. Free-throw accuracy (percentage-point difference)

Specification	N (T1/T2)	T1 estimate [95% CI]	SE	Wald	p	T2 estimate [95% CI]	SE	Wald	p
Primary mixed model	240/240	6.70 [4.30, 9.10]	1.22	5.47	$< .001$	6.10 [3.70, 8.50]	1.22	4.98	$< .001$
Baseline-adjusted ANCOVA	234/229	6.62 [4.18, 9.06]	1.24	5.32	$< .001$	6.04 [3.60, 8.48]	1.24	4.85	$< .001$
Multiple imputation	240/240	6.59 [4.21, 8.97]	1.21	5.43	$< .001$	6.02 [3.65, 8.39]	1.21	4.98	$< .001$
Complete case	227/227	6.75 [4.27, 9.23]	1.27	5.33	$< .001$	6.13 [3.64, 8.62]	1.27	4.83	$< .001$
Protocol-compliant	230/230	6.84 [4.35, 9.33]	1.27	5.38	$< .001$	6.18 [3.67, 8.69]	1.28	4.83	$< .001$
Attendance-adjusted	240/240	6.61 [4.22, 9.00]	1.22	5.42	$< .001$	6.03 [3.64, 8.42]	1.22	4.95	$< .001$
Exposure-adjusted	240/240	6.55 [4.14, 8.96]	1.23	5.33	$< .001$	5.98 [3.57, 8.39]	1.23	4.86	$< .001$
Course-section adjusted	240/240	6.65 [4.20, 9.10]	1.25	5.32	$< .001$	6.08 [3.61, 8.55]	1.26	4.82	$< .001$

Panel B. Technique quality (scale-point difference)

Specification	N (T1/T2)	T1 estimate [95% CI]	SE	Wald	p	T2 estimate [95% CI]	SE	Wald	p
Primary mixed model	240/240	1.74 [1.14, 2.34]	0.31	5.68	$< .001$	1.63 [1.01, 2.25]	0.32	5.15	$< .001$
Baseline-adjusted ANCOVA	234/229	1.70 [1.08, 2.32]	0.32	5.37	$< .001$	1.58 [0.95, 2.21]	0.32	4.92	$< .001$
Multiple imputation	240/240	1.71 [1.12, 2.30]	0.30	5.68	$< .001$	1.60 [1.00, 2.20]	0.31	5.23	$< .001$
Complete case	227/227	1.76 [1.14, 2.38]	0.32	5.56	$< .001$	1.66 [1.02, 2.30]	0.33	5.08	$< .001$
Protocol-compliant	230/230	1.79 [1.16, 2.42]	0.32	5.57	$< .001$	1.67 [1.02, 2.32]	0.33	5.04	$< .001$
Attendance-adjusted	240/240	1.72 [1.12, 2.32]	0.31	5.62	$< .001$	1.61 [1.00, 2.22]	0.31	5.17	$< .001$
Exposure-adjusted	240/240	1.70 [1.09, 2.31]	0.31	5.46	$< .001$	1.59 [0.98, 2.20]	0.31	5.11	$< .001$
Course-section adjusted	240/240	1.73 [1.11, 2.35]	0.32	5.47	$< .001$	1.62 [0.99, 2.25]	0.32	5.04	$< .001$

Note: Accuracy effects are percentage-point differences; technique effects are scale-point differences. Mixed-model N denotes the randomized ITT analysis population contributing baseline and/or post-baseline repeated observations; ANCOVA N is occasion-specific. Wald statistics are shown to make the robustness analyses numerically auditable alongside SE, 95% CI, and p values.
